# *KCNQ1 *gene polymorphisms are associated with lipid parameters in a Chinese Han population

**DOI:** 10.1186/1475-2840-9-35

**Published:** 2010-08-11

**Authors:** Zhong Chen, Quanzhong Yin, Genshan Ma, Qi Qian

**Affiliations:** 1 Department of Cardiology, the Affiliated ZhongDa Hospital and Institute of Cardiovascular Disease of Southeast University, Nanjing 210009, P.R. China; 2 Department of Cardiology, the Affiliated Jiangyin Hospital of Southeast University, Jiangyin 214400, P.R.China

## Abstract

**Background:**

Four single nucleotide polymorphisms (SNPs) (rs2237892, rs2237895, rs2237897, and rs2283228) in *KCNQ1 *are reported to be associated with type 2 diabetes mellitus (T2DM), possibly caused by a reduction in insulin secretion and higher fasting glucose, but the results are inconsistent. We investigated whether these 4 genetic markers are associated with serum lipid metabolism in a middle-aged Chinese Han population.

**Methods:**

We enrolled 398 consecutive patients, including 180 with premature coronary artery disease (CAD) (male < 55 years, female < 65 years) and 218 controls without documented CAD. All subjects were genotyped for 4 SNPs by using the ligase detection reaction method. Fasting blood sugar (FBS) and plasma concentrations of total cholesterol, triglycerides (TG), high-density lipoprotein cholesterol (HDL-C), low-density lipoprotein cholesterol (LDL-C), apolipoprotein A1(apo A1), and apolipoprotein B (apo B) were determined by standard biochemical methods. Main anthropometric and metabolic characteristics are analyzed among 3 genotypes at rs2283228, rs2237895, rs2237897, or rs2237892 in *KCNQ1*.

**Results:**

The 3 genotypes AA, AC, and CC were present in rs2283228 and rs2237895, and the 3 genotypes CC, CT, and TT were present in rs2237897 and rs2237892. The minor genotypes CC at rs2283228 and TT at rs2237892 were associated with higher levels of TG (*P *= 0.007 and 0.026, respectively). Furthermore, subjects with the CC genotype at rs2283228 had lower levels of HDL-C and apo A1 than in the other 2 genotype groups (*P *= 0.052 and 0.055, respectively). No other associations were detected between these 4 SNPs and FBS or other lipid parameters.

**Conclusions:**

Our data suggest that rs2283228 and rs2237892 in *KCNQ1 *are associated with lipid metabolism in a middle-aged Chinese Han population.

## Background

*KCNQ1*, which is located at chromosome 11p15.5 and near a candidate region at 11p13-p12 and mainly encodes a protein for a voltage-gated potassium channel required for the repolarization phase of the cardiac action potential, has been proved to be associated with type 2 diabetes mellitus (T2DM). Studies done by different groups show that some common single nucleotide polymorphisms (SNPs) in *KCNQ1*, acting as T2DM susceptibility genes, exist in different ancestral groups including in the Asian populations of the Japanese, Korean, and Chinese in Hong Kong and Shanghai [1-5]. Among these are the 4 common SNPs rs2237892, rs2237895, rs2237897, and rs2283228 that were well replicated. However, controversies still exist in which these SNPs in *KCNQ1 *are not implicated as T2DM susceptibility genes in individuals of European descent [6,7] or in a middle-aged population from East China [8].

Owing to the lack of consistent replication of these results in case-control studies in different study populations, it remains to be determined whether the results derived from Western societies are internationally applicable to East Asian races or specifically the Chinese Han population, or whether genetic background can cause different clinical phenotypes of the same disease as coronary artery disease (CAD) or T2DM.

Only recently have scholars demonstrated that the increased risk for T2DM linked to SNPs in *KCNQ1 *is likely to be caused by a reduction in insulin secretion, higher levels of fasting glucose or HbA1c [5,9,10], which implies that the *KCNQ1 *variants may play a major physiological role in the metabolism and dynamic balance of blood glucose. Furthermore, some other gene polymorphisms such as hepatocyte nuclear factor-1 homeobox A (HNF1A) gene polymorphisms [11], PPARgamma gene C161T polymorphism [12] and adiponectin receptor-2 gene variations [13] have also been proved to be connected with diabetes and cardiovascular disease.

Given that it is still unknown whether these 4 SNPs in *KCNQ1 *are associated with plasma lipid parameters besides their inconsistent association with T2DM [1-10], to fill this knowledge gap, we aimed to determine whether these 4 common SNPs at *KCNQ1 *loci would correlate with plasma lipid parameters in a middle-aged Chinese Han population living in East China. The results will help solve this apparent controversy and provide new evidence to elucidate the mechanism of *KCNQ1 *variants on the risk of T2DM and CAD.

## Methods

### Study population samples

The study population has been previously described [8]. Briefly, from January 2003 to August 2008, 398 patients undergoing coronary angiography (CAG) for chest discomfort or suspected CAD were enrolled in this study, including 180 patients (men age 32-54 years and women age 39-64 years) with documented premature CAD [14] and 218 subjects without coronary stenosis, acting as controls. CAD was defined as a significant coronary stenosis (≥ 50%) in at least one of the 3 main coronary arteries or their major branches (branch diameter ≥ 2 mm) assessed by CAG or having experienced a myocardial infarction (MI) defined according to World Health Organization criteria. All patients with type 1 diabetes mellitus, congenital heart disease, syndrome X, severe liver or kidney disease, or noncoronary artery thrombotic disease were excluded from this study. The study was approved by the Medical Ethics Committee of the Affiliated ZhongDa Hospital of Southeast University. Before enrollment, the trial information was explained carefully to each patient, and subsequently written informed consent was obtained from all participants.

### Coronary angiography

All participants underwent CAG during hospitalization. The grade of the coronary stenosis and CAD diagnosis were judged by 2 cardiologists unaware of this study.

### Determination of parameters and risk factors

At the time of enrollment, background data, such as sex, age, height, body weight, and risk factors including hypertension, T2DM, family history of CAD, and smoking status were collected from each subject. Anthropometric measurements and blood pressure determination were performed according to standard protocols. Blood was collected and plasma concentrations of total cholesterol (TC), triglycerides (TG), high-density lipoprotein cholesterol (HDL-C), and low-density lipoprotein cholesterol (LDL-C) were analyzed as previously described [15]. Levels of apolipoprotein A1 (apo A1), apolipoprotein B (apo B), fasting blood sugar (FBS), and insulin were determined by standard biochemical methods using a chemistry analyzer (Beckman Coulter Synchron clinical system LX20). Insulin resistance was calculated from fasting insulin and glucose by using the homeostasis model assessment method [16].

Determination of hypertension, T2DM, family history of CAD, and smoking status has been previously described [8]. Body weight and height were measured with individuals wearing light clothing, and body mass index (BMI) is expressed as weight in kilograms divided by the square of the height in meters.

### DNA extraction and genotyping

Genomic DNA extraction and genotyping of 4 SNPs in intron 15 of *KCNQ1 *have been described previously [8]. Genotype distributions obeyed the Hardy-Weinberg equilibrium (*P *> 0.05).

### Statistical analyses

Statistical analyses were conducted using SPSS 15.0 software. Continuous data are expressed as mean ± SD, and categorical variables are expressed as frequencies. Analyses of continuous data and categorical variables among 3 different groups according to the genotypes of these 4 SNPs at *KCNQ1 *were determined by one-way ANOVA or chi-square test. Two-tailed *P-*values *<*0.05 were considered as significant.

## Results

### Analysis of baseline characteristics in patients among 3 different genotypic groups (Table 1 and Table 2)

**Table 1:** Comparisons of anthropometric and metabolic characteristics among 3 genotypes at rs2283228

	Genotype			*P*
rs2283228	AA	AC	CC	
Numbers	156	191	51	
Sex, male (n/%)	77/49.3	90/47.1	27/52.9	0.746
Age, years	51.67 ± 5.50	51.28 ± 5.47	52.53 ± 5.72	0.836
Hypertension, n/%	83/53.2	121/63.3	31/60.7	0.155
Type 2 diabetes mellitus, n/%	21/13.5	29/15.2	7/13.7	0.894
Family history of CAD, n/%	64/41.0	73/38.2	17/33.3	0.609
Smokers, n/%	53/33.9	69/36.1	17/33.3	0.887
BMI, kg/m^2^	24.29 ± 3.46	24.93 ± 3.81	24.33 ± 3.00	0.227
FBS, mmol/L	5.73 ± 1.72	5.57 ± 0.98	5.87 ± 1.86	0.408
Insulin, mU/L	14.31 ± 11.87	13.16 ± 9.84	17.32 ± 13.08	0.351
HOMA-IR	3.64 ± 0.59	3.56 ± 0.57	4.36 ± 0.83	0.138
TC, mmol/L	4.65 ± 0.93	4.39 ± 0.86	4.63 ± 0.78	0.116
TG, mmol/L	1.53 ± 0.76	1.50 ± 0.80	2.03 ± 0.84	0.007
LDL-C, mmol/L	2.82 ± 0.80	2.71 ± 0.74	2.74 ± 0.84	0.604
HDL-C, mmol/L	1.21 ± 0.30	1.14 ± 0.25	1.07 ± 0.22	0.052
Apo A1, g/L	1.15 ± 0.18	1.11 ± 0.19	1.08 ± 0.16	0.055
Apo B, g/L	0.87 ± 0.25	0.86 ± 0.25	0.97 ± 0.23	0.115

Data are mean ± SD, or number (%), as appropriate.

*P *is the significance level of comparison of clinical and plasma parameters among 3 groups by one-way ANOVA or chi-square test.

Apo A1, apolipoprotein A1; Apo B, apolipoprotein B; BMI, body mass index; CAD, coronary artery disease; FBS, fasting blood sugar; HDL-C, high-density lipoprotein cholesterol; HOMA-IR, homeostasis model assessment-insulin resistance; LDL-C, low-density lipoprotein cholesterol; SNPs, single nucleotide polymorphisms; TC, total cholesterol; TG, triglyceride.

**Table 2:** Comparisons of anthropometric and metabolic characteristics among 3 genotypes at rs2237892

	Genotype			*P*
rs2237892	CC	CT	TT	
Numbers	189	168	41	
Sex, male (n/%)	92/48.7	81/48.2	21/51.2	0.942
Age, years	51.04 ± 5.48	51.96 ± 5.50	52.64 ± 5.39	0.864
Hypertension, n/%	106/56.1	101/60.1	28/68.3	0.330
Type 2 diabetes mellitus, n/%	27/14.3	24/14.3	6/14.6	0.998
Family history of CAD, n/%	73/38.6	63/37.5	18/43.9	0.752
Smokers, n/%	63/33.3	61/36.3	15/36.6	0.818
BMI, kg/m^2^	24.34 ± 3.35	24.89 ± 3.94	24.59 ± 3.00	0.362
FBS, mmol/L	5.73 ± 1.61	5.51 ± 0.94	6.04 ± 2.01	0.120
Insulin, mU/L	13.70 ± 11.28	13.43 ± 10.02	18.66 ± 13.04	0.232
HOMA-IR	3.68 ± 0.69	3.51 ± 0.59	4.78 ± 0.81	0.437
TC, mmol/L	4.60 ± 0.90	4.41 ± 0.90	4.75 ± 0.66	0.157
TG, mmol/L	1.60 ± 0.83	1.48 ± 0.74	2.01 ± 0.87	0.026
LDL-C, mmol/L	2.77 ± 0.78	2.72 ± 0.77	2.85 ± 0.82	0.759
HDL-C, mmol/L	1.19 ± 0.29	1.14 ± 0.25	1.09 ± 0.25	0.178
Apo A1, g/L	1.14 ± 0.18	1.08 ± 0.19	1.12 ± 0.18	0.095
Apo B, g/L	0.88 ± 0.26	0.85 ± 0.25	0.92 ± 0.21	0.072

Abbreviations see Table 1.

The 3 genotypes AA, AC, and CC are in rs2283228 and rs2237895, and the 3 genotypes CC, CT, and TT are in rs2237897 and rs2237892. When all study subjects were divided into 3 groups as the AA, AC, and CC group in rs2283228 and rs2237895, or the CC, CT, and TT group in rs2237897 and rs2237892, we found that 3 groups were well matched for mean age and gender composition. Subjects with the CC genotype in rs2283228 and TT genotype in rs2237892 had significantly higher levels of TG compared with the other 2 groups (*P *< 0.05), but this was not the case in subjects with the CC genotype in rs2237895 or TT genotype in rs2237897 (data not shown).

Overall in the study population, no significant differences were detected in the prevalences of hypertension, T2DM, family history of CAD, smoking, and average values of BMI, FBS, insulin, TC, LDL-C, and homeostasis model assessment of insulin resistance (HOMA-IR) among 3 groups according to 3 different genotypes at 4 SNPs in *KCNQ1 *(all *P *> 0.05).

Despite the lack of association between genotypes and TC, LDL-C, and apo B, concentrations of HDL-C and apo A1 in the CC group in rs2283228 were lower at the borderline significance level compared with the other 2 groups (*P *= 0.052 and 0.055, respectively). No other associations were detected between concentrations of HDL-C, apo A1, and apo B and SNPs at rs2237892, rs2237895, or rs2237897 (all *P *> 0.05).

## Discussion

In the present study, for the first time, we report a strong association between rs2283228 and rs2237892 in *KCNQ1 *and lipid metabolism in a middle-aged Chinese Han population, which adds new data to this study issue.

Better understanding and preventive strategies for the morbidity and mortality related to T2DM are compelling. Recently, some susceptibility genes for T2DM have been reported [1-4], and potential mechanisms have also been elucidated [5,9,10]. However, inconsistent results still exist [6-8], and other mechanisms could not be fully excluded.

In the study by Tan JT et al [9], a total of 3 734 participants (2 520 Chinese, 693 Malay, and 521 Asian Indians) living in Singapore were enrolled, among which 1 881 Chinese subjects with normal glucose tolerance served as controls, and the reference population had an age range from 18 to 69 years. In the study by Liu Y et al [5], there were more women in both T2DM cases (women, 1 127; men, 785) and controls (women, 1 406; men, 635) and higher average age [(63.9 ± 9.5) years *vs*. (58.1 ± 9.4) years] than in our study. For the present study, all participants were aged 32 to 64 years and were hospitalized for CAD screening, among which 14.3% of subjects were diagnosed with T2DM, 180 subjects (45.2%) had CAD, and 218 subjects (54.8%) were non-CAD controls [8]. Young CAD patients usually have a different profile of major coronary risk factors [17]. Obviously, the differences in enrollment criteria and population characteristics account for the discrepancies between these studies. Meanwhile, differences in genetic inheritance among the races, lifestyles, public health insurance, and a system of free access to hospitals might contribute to the available subjects enrolled in our study.

In the current study, subjects in 3 groups according to different genotypes at rs2283228, rs2237895, rs2237897, and rs2237892 were well matched for mean age and gender composition, and no significant differences were detected in the prevalences of hypertension, T2DM, family history of CAD, smoking and values of BMI, FBS, insulin, TC, LDL-C, and HOMA-IR.

More recently, emerging evidence supports TG as a risk factor for CAD [18,19]. Patients with T2DM are more likely to have higher levels of TG and lower levels of HDL-C. Our study thus furthermore helps settle the issue of whether the variants in *KCNQ1 *are related to lipid metabolism. In the present study, we demonstrated that an association exists between CC genotype in rs2283228 and TT genotype in rs2237892 and higher levels of TG. Further fine mapping of SNPs in *KCNQ1*, especially within the LD block containing rs2283228 or rs2237892, may allow the eventual identification of the causal variant. Meanwhile, in the study by Giuffrida et al, though HNF1A I27L polymorphism did not differ significantly among late-onset autosomal dominant diabetes mellitus, classical T2DM and normoglycemic controls, it was found to be associated with risk of hypertriglyceridemia [11]. In patients with CAD combined with T2DM, P PARgamma C161 > T genotypes are associated with levels of TG and apo B but not glucose metabolism, with CC homozygote carriers having significantly higher levels of TG and apo B than those in T allele carriers [12]. Large prospective studies have identified HDL-C as a strong, independent, inverse predictor of risk of CAD [20,21]. Patients with higher levels of HDL-C and CAD had a similar or lower prevalence of traditional CAD risk factors compared with patients with normal HDL-C levels and CAD [22]. In our study, subjects with CC genotype in rs2283228 had lower levels of HDL-C and apo A1.

Some limitations of the present study need to be acknowledged. First, our study population comprised only those who have been admitted to the hospital for CAD screening and diagnosis, so it's more representative of patients in a cardiac center and might not be representative of the general population. Additionally, our study sample is predominantly yellow; thus, the results may not be generalizable to other ethnic groups in which disparities in population composition, geographical, and ethnic backgrounds may exist. It is well known that significant differences exist in the frequencies of some genetic variations among different ethnic groups and geographical regions. The fact is that these novel observations have not been previously found in other ethnic groups.

## Conclusion

Our novel findings that the risk alleles of rs2283228 and rs2237892 within the *KCNQ1 *gene are associated with levels of TG, HDL-C and apo A1 in a middle-aged Chinese Han population are encouraging. Though the causal effects of four common SNPs (rs2237892, rs2237895, rs2237897, and rs2283228) in *KCNQ1 *on T2DM are controversial, our present study, for the first time, provide new evidence that these variants have an effect on lipid metabolism. Further studies are needed to firmly replicate these promising findings in other populations and to fully delineate the role of *KCNQ1 *and its related pathways in the pathogenesis of T2DM and CAD.

## Abbreviations

Apo A1: apolipoprotein A1; Apo B: apolipoprotein B; BMI: body mass index; CAD: coronary artery disease; CAG: coronary angiography; FBS: fasting blood sugar; HDL-C: high-density lipoprotein cholesterol; HNF1A: hepatocyte nuclear factor-1 homeobox A; HOMA-IR: homeostasis model assessment-insulin resistance; LDL-C: low-density lipoprotein cholesterol; SNPs: single nucleotide polymorphisms; TC: total cholesterol; TG: triglyceride; T2DM: type 2 diabetes mellitus.

## Competing interests

The authors declare that they have no competing interests.

## Authors' contributions

ZC conceived the study, designed and performed the study experiments, analyzed the data and interpreted the results and wrote the manuscript. QZY, GSM and QQ interpreted the results, participated in the writing of the manuscript. All authors read and approved the final manuscript.

## Author information

^1^Department of Cardiology, the Affiliated ZhongDa Hospital and Institute of Cardiovascular Disease of Southeast University, NO.87 Dingjiaqiao, Nanjing 210009, P.R.China

^2^Department of Cardiology, the Affiliated Jiangyin Hospital of Southeast University, Jiangyin 214400, P.R.China
